# The African Organisation for Research and Training in Cancer and its conferences: a historical perspective and highlights of the Ninth International Conference, Durban, South Africa, 21–24 November 2013

**DOI:** 10.3332/ecancer.2014.396

**Published:** 2014-02-03

**Authors:** Christopher KO Williams, D Cristina Stefan, Fiona Rawlinson, Kenneth Simbiri, Sam M Mbulaiteye

**Affiliations:** 1 Hematology Oncology Consultancy, Port Angeles, WA 98362, USA and Fred Hutchinson Cancer Research Center/University of Washington Center for AIDS Research (CFAR), Seattle, WA 98195, USA; 2 Department of Paediatrics, University of Stellenbosch, Stellenbosch 7600, Cape Town, South Africa; 3 University of Wales College of Medicine, Velindre Hospital, Cardiff CF14 2TL, Wales, UK; 4 Department of Microbiology and Immunology, 2021A Weiskotten Hall, Upstate Medical University, Syracuse, NY 13210, USA; 5 National Institutes of Health/NCI/DCEG, Infections and Immunoepidemiology Branch, 9609 Medical Center Dr, Rm. 6E118 MSC 9706, Bethesda MD 20892–9704, USA

**Keywords:** cancer control in Africa, AORTIC cancer conference, EMBLEM, WHO Africa NCD

## Abstract

The objectives of the African Organisation for Research and Training in Cancer (AORTIC), both at its inception in the early 1980s, and at its reactivation in 2000 following a decade of inactivity, included bringing the products of decades of advances in cancer research to African populations through international collaboration. The historical perspective provided in this report illustrates progress in achieving these objectives through successive continent-wide activities over a period of 30 years, culminating in the organisation’s most recent conference held in Durban, South Africa, 21–24 November 2013. The constant growth in the number of attendants and increasing diversity of the nations of their origin are consistent with advances, whereby the number of participants and the nations of their origin have grown from 24 in 1983 to almost 1000 in 2013, and from 14 to 70, respectively. While earlier AORTIC conferences used to assume the atmosphere of ‘jamborees’, more recent ones have morphed to problem-solving events, with the concerted collaboration of international organisations, including the World Health Organisation (WHO), International Union Against Cancer (UICC), the Africa Union (AU), the US National Cancer Institute (NCI), the International Psycho-Oncology Society (IPOS), and others. The topics of discussion at the Ninth AORTIC International Conference on Cancer in Africa in Durban were those of paramount importance for low- and middle-income countries: childhood cancers, cancers of the cervix, breast, and prostate, as well as cancer care challenges resulting from ignorance, neglect, and economic deprivation. The role of environmental factors that underlie Burkitt’s lymphoma was the subject of the Epidemiology of Burkitt Lymphoma in East-African

Children and Minors Workshop, highlighting the NCI research programme in East Africa, while the Workshop on Cost Effectiveness of Treatment of Cancer in Africa surmised that treating childhood cancers is affordable in Africa in spite of widespread economic deprivation. WHO representatives emphasised the organisation’s commitment to the global control of non-communicable diseases (NCDs), including cancer, and promoted the new initiatives for the control of cervical cancer, one of the commonest and deadliest cancers in adult Africans. AU representative proffered the principles of ‘demographic dividends’ for Africa to be able to tackle its burden of NCDs. UICC, represented by its President, provided guidelines for cancer diagnosis and staging, and advised on its effort to improve global access to radiotherapy, especially in Africa, while IPOS led the discussions on mitigating the suffering that is associated with the late presentation of cancer in the region. Oral and poster presentations from various parts of the continent indicate the growth of basic science of cancer in the region, with studies revealing regional diversity in the frequencies of the triple-negative breast cancer. They also suggest a need for genome-wide association studies as well as the evaluation of single nucleotide polymorphisms that may be responsible for variable susceptibility in breast and prostate cancer in people of African descent. Finally, the AORTIC leadership announced its plan for the advancement of cancer control by intensifying cancer advocacy at all levels of governance in the region.

## Historical perspectives

A report on a conference of a cancer organisation in a region of the world where the preventable diseases of neglect and poverty inundate the health care system is likely to raise eyebrows. However, with the recent advisory by the World Health Organisation (WHO) that noncommunicable diseases (NCDs) (UNGASS 2011), including cancer, are a threat to all countries, such an activity of a regional cancer control organisation should not be all that surprising.

The history of the African Organisation for Research and Training in Cancer (AORTIC) has previously been documented [1–2]. The founding of AORTIC was the product of an informal lunch break encounter of four acquainted conference participants during the 13th International Cancer Congress held in Seattle, Washington, USA, 8–15 September 1982. They were Dr James Holland of New York City, USA, Dr Victor A Ngu, Dr Toriola F Solanke, and Dr Christopher KO Williams, the latter three being colleagues from the University College Hospital, Ibadan, Nigeria. The subject of discussion that fired the desire to found the organisation was the indignation at the failure of post-independence African rulers to maintain the status of excellence in medical research in places, such as Ibadan, Nigeria, and Kampala, Uganda, achieved largely through the British colonial educational influence that enable these centres to play a significant role in the early days of the global struggle against cancer. The group committed to holding an inaugural conference of the new organisation in Africa within a year.

### Inaugural meeting and the first decade of AORTIC

The inaugural meeting of AORTIC was held in the city of Lome, Togo, 22–23 July 1983. Twenty-four physicians attended it from 14 countries, including Benin, Cameroon, Republic of Congo, Ivory Coast, Kenya, Liberia, Malawi, Mali, Nigeria, Senegal, Sweden, the United States of America, Uganda, and Upper Volta (now renamed Burkina Faso). Dr Jan Stjernsward of Sweden represented the WHO’s headquarters, while Dr Charles Olweny of Uganda and Dr Papa Toure of Senegal represented the African WHO office.

### The Second AORTIC Meeting

This was on 11 November 1985 at the African Headquarters of the WHO, Brazzaville, Congo, and was attended by 61 participants from various African countries.

### The Third AORTIC Meeting

This was held 30 October–3 November 1989 at the Hotel Intercontinental, Kinshasa, Zaire (now renamed Democratic Republic of Congo). There were almost 100 participants, and 80 abstracts were published; about 70 presentations were made in English or French. Participants came from 28 countries, including USA, Canada, Sweden, and France. Regrettably, this was to be the last AORTIC scientific meeting, until its subsequent reactivation in 2000 (see below).

### AORTIC became defunct by the end of 1990

After the third AORTIC conference of November 1989, the organisation rapidly unraveled, apparently as a result of several events, including emigration of its leaders out of Africa in the midst of rapid deterioration of political and socio-economic conditions on the continent.

### Reactivation of AORTIC

By the end of the 1990s, conditions were emerging primarily in the developed world, but also in Africa, which presaged greater chances of success for a transcontinental organisation such as AORTIC than in the 1980s. These included improved communication following the emergence of the communication highway and the Internet revolution, improved political climate, especially, the potential role of South Africa after the demise of apartheid, and of Nigeria, after the demise of military dictatorship. Furthermore, the emerging worldwide cancer burden in relation to the new realities of the acquired immunodeficiency syndrome (AIDS) indicated a need for cancer control in Africa.

### Creation of AORTIC International

AORTIC International was created as an instrument for the resuscitation of AORTIC. The objectives of AORTIC International at its creation included assisting African cancer care professionals and scientists find their rightful place in the world so that they could bring the products of four decades of advances in cancer research to Africans. The body decided to meet at major North American cancer meetings, such as the American Association for Cancer Research (AACR) and the American Society of Clinical Oncology (ASCO) annual meetings. Important decisions made at these meetings included:

- drafting of a constitution for AORTIC;
- incorporation of AORTIC in the US for financial sponsorship, but also additional in other countries;
- creating an AORTIC web site (http://www.aortic.org);
- organising an AORTIC meeting in Africa;
- promotion of South African participation in AORTIC;
- outreach to international organisations for potential support, including the National Cancer Institute (NCI) of USA, the WHO, International Union Against Cancer (UICC), American Cancer Society, AACR, ASCO, and others.

### Meetings of AORTIC International 2001–2003

Between 2001 and 2003, the meetings of AORTIC International were geared towards the repatriation of AORTIC to Africa. Thus, AORTIC International (now doing business as AORTIC North America) was incorporated in New York, USA. The status facilitates access to funding by public donation, which is currently one of the main sources of funding of AORTIC programmes in Africa.

## Repatriation of AORTIC

### The Fourth AORTIC International Conference on Cancer in Africa

The repatriation of AORTIC to Africa took place at the 4th AORTIC Conference on Cancer in Africa, held at the Labadi Beach Hotel in Accra, Ghana, 6–10 October 2003. The conference provided the opportunity of identifying Africa-based leaders that could take on the operation of AORTIC from within Africa, with the intellectual guidance and financial support of AORTIC International. Dr Seth Ayettey of Ghana emerged as the President, while Dr Lynette Denny of South Africa was elected as the Secretary/Treasurer.

### The Fifth AORTIC International Conference on Cancer in Africa

This was held in Dakar, Senegal, 14–16 November 2005 under the patronage of the President of Senegal, who not only attended the opening ceremony, but also gave an inspiring speech on his vision of cancer control in Africa. The Dakar conference was a milestone for the commitment by an African head of state in support of cancer control on the African continent.

### The Sixth AORTIC International Conference on Cancer in Africa

This was held 25–28 October 2007 in Cape Town, South Africa. It was a milestone for the introduction into AORTIC meetings of modern commercial conference organisational facilities. The 25th Anniversary of AORTIC coincided with the 6th AORTIC Conference in Cape Town in 2007, and it was celebrated in a colourful manner, with a parade of the national flags of AORTIC members, and entertainment by a group of local traditional dancers.

### The Seventh AORTIC International Conference on Cancer in Africa

This was held 11–14 November 2009 in Dar es Salaam, Tanzania, and was attended by Mr Jakwaya Kikwete, the President of Tanzania, who committed to support in involving fellow African Heads of State in cancer control in Africa.

### The Eighth AORTIC International Conference on Cancer in Africa

The 8th AORTIC conference took place in Cairo from 30 November to 3 December 2011, in the midst of political turmoil in the host country. Unfortunately, several participants, including scheduled speakers, exhibitors, and sponsors of events, most of whom were located in Europe and USA, cancelled their participation out of security concerns, thus leading to a significantly diminished event. In spite of this, the meeting turned out to be of significant success, attended by 300 participants, from 51 nations.

### The Ninth AORTIC International Conference on Cancer in Africa

The meeting was held at the magnificent International Conference Centre in Durban, South Africa, from 21–24 November 2013. There were 900 participants from 70 countries, thus, making it the most successful of all AORTIC conferences. Participants came from USA, Europe, Africa, and Asia, including a delegation from China.

Pre-conference workshops took place from 20–21 November 2013 involving the following organisations/Topics:

1. AORTIC—International Psycho-Oncology Society Academy.
2. Epidemiology of Burkitt Lymphoma in East-African Children and Minors (EMBLEM) and selected NCI funded research on Burkitt’s lymphoma.
3. Cervical cancer prevention I.
4. Cervical cancer prevention II.
5. Cancer Survivors Forum.
6. HPV Cervical Cancer Network in Francophone Africa.
7. Best practice on prostate cancer.
8. Cancer and sexual health.
9. NCDS African outcomes.
10. The Case for Cancer Registry in Africa.
11. AORTIC/IUBMB/NCI (USA) Oesophageal Cancer Symposium.
12. Cancer Advocacy Workshop and Expo.
13. BIG CAT Grants I.
14. The African perspective on cancer control and NCDs.
15. MD Anderson Global Initiative for Cancer Care in Africa.

Several of these workshops were preludes to regularly scheduled conference sessions.

## Highlights of the Ninth International Conference, held in Durban, South Africa, 21–24 November 2013

### The African perspective on cancer control and NCDs

A number of presentations made at this session, held on 21 November 2013, helped set the tone for the conference. Dr Jean-Marie Dangou, a pathologist from Senegal currently based at the WHO African Regional Headquarters, outlined the current position of his organisation on global NCD in general, and cancer in particular. The importance of increased emphasis on these diseases in Africa, a region that hitherto has been dominated by communicable diseases, was stressed.

Dr A Olajide, a Nigerian economist currently based at the Africa Union (AU) Headquarters in Addis Ababa, made a presentation on ‘Demographic dividends in Africa’, in which he highlighted the demographic changes among the ‘Asian Giants’, with special reference to China, and how this is impacting on the overall quality of life in general, and the control of NCDs in general. He pointed out that the African region lags behind these countries in demographic dividends, and hence lacks the prospects of improvement in its quality of life and the capacity to address the challenges of NDC in general, and cancer in particular.

## Report according to disease and subject type

### Burkitt’s lymphoma

#### Workshop on the EMBLEM and Selected NCI-Funded Research on Burkitt’s Lymphoma

The coverage of this important childhood cancer of African children started with the Workshop on EMBLEM as one of the pre-conference workshops. It was held on 20 November 2013 and was chaired by Dr Kishor Bhatia (USA), Dr Sam Mbulaiteye (USA), and Dr Kenneth Simbiri (USA). The workshop, which included presentations from 15 scientists representing 13 institutions, highlighted work on Burkitt’s lymphoma research in East Africa covering the topics of epidemiology, clinical outcomes, and immunology studies of BL, principles of cancer registration, capacity building, scientific publication, and collaborative research through a consortium. The workshop commenced with reports about the EMBLEM Study, including description of the study design, objectives and timeline. EMBLEM is a collaborative multicentric study led by investigators at the U.S. NCI (Dr Mbulaiteye is the principal investigator) working with local co-principal investigators in six rural regions in Uganda, Tanzania, and Kenya. It will evaluate the role of:

- repeated malaria infections in endemic Burkitt’s lymphoma;
- malaria genetic variants in Burkitt’s lymphoma;
- Epstein–Barr virus (EBV) genetic variants in Burkitt’s lymphoma;
- host genetics in Burkitt’s lymphoma.

The specific hypotheses that will be tested are:

- carriage of genetic markers that influence resistance to malaria is associated with risk of Burkitt’s lymphoma;
- carriage of rare EBV genetic variants is associated with increased risk for Burkitt’s lymphoma;
- host genetic variants are associated with risk of Burkitt’s lymphoma.

In addition to generating unique data and sample repository for novel studies of the aetiology and biology of Burkitt’s lymphoma, EMBLEM serves as unique model for introducing equitable sustainable high-quality research in Africa and improving capacity for pathology and clinical studies in paediatric cancers.

EMBLEM progress reports were presented by co-investigators from Uganda (St. Mary’s Hospital Lacor [Gulu], Kuluva Hospital [Arua]) (co-principal investigator: Dr Martin D Ogwang), Kenya (Homabay District Hospital [Nyanza], Webuye District Hospital [Western] (co-principal investigator: Dr Constance Tenge), and Tanzania (Bugando Medical Centre [Mwanza], Shirati Hospital [North Mara] (co-principal investigator: Dr Esther Kawira). They all described the setting up of well-functioning coordinated infrastructure for health communication about Burkitt’s lymphoma, case spotting, pathology diagnosis, biomedical sample collection, storage, and handling in what appeared to be rural hospital settings with otherwise basic facilities. The possibility that the described infrastructure could serve as examples for setting up of biomedical research facilities in similar settings in other parts of sub-Sahara Africa (SSA) was raised during the discussion period. Additionally, the multicentric model was suggested as a model for establishing a consortium for research on Burkitt’s Lymphoma Research in Africa to strengthen and magnify the work of any individual research team and hasten the day when research findings result in better prevention, care, and cure for BL patients.

Of significance was the granting by AORTIC committee for EMBLEM to have a full-day pre-conference meeting enabling the sharing of more work taking place in different regions. The presentation by Dr Kishor Bhatia on current molecular studies in BL and possible directions that we should focus on with the current technology was challenging. The conference had broad coverage of Cancer Registry in Africa, but Dr Robert Newton brought it home with a focus on BL in Africa that addressed the challenges and way forward. Diagnosis of BL is important, but so is treatment, and this was eloquently addressed by the studies of Dr Cristina Stefan (South Africa), Dr Fred Okuku (Uganda), Dr Juliana Otieno (Kenya), and Dr Ann Moormann (USA). Dr Kenneth Simbiri (USA) presented on the need to look at other oncogenic herpes viruses and infectious agents as their contribution to BL and other virally associated cancers have not been fully investigated a paradigm shift worth looking into considering the plethora of infections most Africans are exposed to. Dr Joe Harford (NIH) addressed the very important topic of capacity building in Africa, what the need is, and what can be done to achieve it including grant writing by African researchers. Ms Detra Robinson from Westat Inc. presented on consortium setting, an area that EMBLEM and other cancer groups in Africa will have to build to make sure that success in conquering cancer in Africa is done through sharing of data and information that benefits all. Finally, we had the honour of hearing a presentation by Dr Franco Buonaguro (co-editor with Dr Sam Mbulaiteye of the *Journal Infectious Agents and Cancer*) on publishing. Dissemination of research findings is critical to the progress of science and it is important we become familiar with the requirements and expertise on writing a presentable manuscript.

#### Treatment of endemic (childhood) Burkitt’s lymphoma

In her comment on the place of treatment of childhood cancers in general, Dr Cristina Stefan of the Stellenbosch University in Cape Town, South Africa, stated that childhood cancers are not preventable but are very responsive to treatment, and hence highly curable. Early diagnosis is important, since the earlier the diagnosis, the greater the curability. Reporting on a pre-conference consensus workshop on the cost of treatment of childhood cancers, she indicated that the treatment of these cancers is highly cost effective, and that the cost of treatment should no longer be a reason for not offering treatment, even in poor resource settings (see Dr Stefan’s report on the workshop below).

#### Other reports on the treatment of Burkitt’s lymphoma

At the Free Communication session of 22 November 2013, Dr Dani Kotze of the Stellenbosch University, South Africa presented a six-year retrospective cohort study of 23 patients with HIV/AIDS associated Burkitt lymphoma, leukaemia, or Burkitt-like lymphoma who were treated at the Tygerberg Hospital, an academic medical centre in Cape Town. Most (96%) of the patients were HIV-positive and 82% were treated with the LMB 86 regimen, while 18% received either the hyper-CVAD or Stanford programmes. The median CD4 lymphocyte count was 174 cells/μL (range: 21–535 cells/μL). Ninety-six per cent of patients presented with advanced stages of disease (Ann Arbor stage III or IV). Two-year overall survival was 47.4%. The most frequent causes of death were infection (22%) and disease progression (22%). Treatment non-compliance was apparently a problem, with up to 40% defaulting treatment. Dr Kotze concluded that despite limited resources, outcomes on the study were comparable with international studies using similar chemotherapeutic regimens in HIV positive BL patients of comparable age and disease stage. He suggested that monitoring and prompt management of treatment toxicity and ensuring regular follow-up visits were essential components for improving outcomes in patient outcome. When asked about the tolerability of the intensive treatment regimen hyper-CVAD, Dr Kotze said only one patient received the regimen and that the patient tolerated it well.

At the poster session of 24 November 2013, Dr Kouie Plo of the University Teaching Hospital, Boake, Cote D’Ivoire, reported on his experience in the management of Burkitt’s lymphoma, which he described as the commonest malignancy in Ivorian children, and that late presentation was the norm. In his study, from November 2011 to January 2013, there were 21 children, including 12 females and nine males aged 6–16 years. They were investigated with routine blood work, tumour needle aspiration and smears, abdomen ultrasonography, lumbar puncture with cerebral spinal fluid cytology, and chemistry. BL staging was based on Murphy’s staging system. The treatment consisted in 4–6 cycles of cyclophosphamide: 600 mg/m^2^d1, d3, d5 d7; doxorubicin: 60 mg/m^2^, d7; methotrexate: (LP) and vincristine: 1.5 mg/m^2^ d3; and prednisone: 100 mg/m^2^ d1–d7. CNS prophylaxis was achieved by intrathecal injection of methotrexate 15 mg/m^2^ and prednisone 25 mg weekly. There were five stage I, three stage II, eight stage III, and five stage IV cases. Complete remission occurred in 35% and partial remission in 65%. Ten patients received consolidation and maintenance treatment for 6–12 months. Five patients relapsed, while three others defaulted on chemotherapy. There were three deaths from drug toxicity and severe infection. The high cost of chemotherapy agents constituted one of the difficulties, resulting in treatment non-compliance and abandonment of the patients by their parents/guardians.

In a presentation on the management of Burkitt’s lymphoma at the Obafemi Awolowo University Teaching Hospital, Ile-Ife, Nigeria, a comparison of experience from two periods was provided. Group A were patients treated under a ‘self-sponsored BL programme’ managed between 1987 and 2000, while Group B were those treated between 2004 and 2012 under a ‘sponsored multicentre international study’ [supported by the International Network for Cancer Treatment and Research] using cyclophosphamide, oncovin, and methotrexate (COM) regimen. The objective of this study was to compare treatment outcomes in the two periods. Consenting BL patients enrolled between December 1986 and September 2000 (Group A), and between September 2004 and July 2011 (Group B). Group A had COM/COMP regimens with cytarabine or MTX being given as intrathecal therapy. Group B had COM regimen as first line therapy and a combination of ifosfamide (and mesna), etoposide, and cytarabine as second line for early relapse, with cytarabine and MTX being given as intrathecal therapy. Overall survival (OS) and event-free survival (EFS) were computed with Kaplan–Meier technique for Group B from the date of induction until the patient died or was censored. There was a high default rate of 88% of Group A patients, thus precluding OS and EFS computation. The male to female ratio was 1.8:1, and median ages at onset of nine and eight years were similar for both groups. Thirty-six (16.8%) of Group A patients had no chemotherapy, 21 (9.8%) were lost to toxic deaths within two weeks of admission and 88% of 157 evaluable patients defaulted before analysis (40 and 13 in CR and PR, respectively) and four were alive 6–164 (median, 61) months post-induction therapy. For Group B, OS was 61.4% and 57.5% at 12 and 24 months; and EFS at 12 and 24 months were 58.3% and 53.4%. The presenter concluded that the better survival obtained for Group B patients was due to availability of chemotherapy, supportive therapy, and efficient patient follow-up.

### Report on workshop: ‘Cost Effectiveness of Treating Cancer in Africa’, 21 November 2013, Durban

Prof D Cristina Stefan of the Department of Paediatrics, Stellenbosch University in Cape Town submitted the following report on a workshop on ‘Cost Effectiveness of Treating Cancer in Africa’:

This one-day ground-breaking workshop was the first of its kind to be held in Africa. It was jointly organised by African colleagues from Rwanda, Senegal, South Africa, and Zimbabwe. It was attended by 39 participants from eight African countries, namely Egypt, Ghana, Rwanda, Senegal, Sierra Leone, South Africa, Tanzania, and Zimbabwe, as well by participants from the United States, United Kingdom, France, Canada, Sweden, and Austria. The workshop was opened by Prof DC Stefan (South Africa) and Dr F Ntaganda (Rwanda). The participants were then addressed by Prof JM Dangou, President of WHO Africa, followed by an inspiring address by Prof E Elzawawy, President of AORTIC. Dr F Bray International Agency for Research in Cancer provided an update on the global cancer burden with the emphasis on the situation in Africa, where breast, cervical, lung, and prostate cancers are still the most common overall. Prof DC Stefan put the spotlight on childhood cancer by sketching the status thereof in Africa, as well as by highlighting important aspects and potential strategies in the fight against it. Prof S Horton gave a very enlightening and informative talk on how to calculate the cost and cost effectiveness of cancer, giving easy tips on how to perform a basic cost assessment. This was followed by a number of participants reporting on the cost of cancer treatment in several African countries: Rwanda (Dr F Ntaganda), Senegal (Prof C Moreira of the Groupe Franco Africain d’Oncologie Pédiatrique), Uganda (Dr C Casper), Zimbabwe (Dr I Chitsike), Malawi (Dr S Gupta), and South Africa (Dr A van Zyl). It was very clear that treating a child with cancer in Africa is extremely cost effective.

An interactive discussion involving all participants took place regarding the role of the pharmaceutical companies in curbing the cost of cancer treatment in Africa, following a brief address by Ms E Anfruns from GlaxoSmithKline. It was agreed that pharmaceutical companies could assist by supporting the collection of data regarding the incidence of cancer in Africa, as well as in cost studies. Mr F Peenz from CHOC South Africa informed the audience about the importance of early identification of cancer and the education of the community and health care workers about childhood cancer. Early detection leads to diagnosis of an earlier stage of disease, which will decrease costs of treatment. All participants then took part in a lively discussion about establishing a working group to study the cost effectiveness of treating cancer in Africa and subsequently the African Cancer Economics Net (ACE-net) was founded. The addendum describes the aims, vision, mission, and activities of this new entity. This was a highly successful workshop with attendance of key people, which has led to the formation of a working group that will work towards gathering more cost data regarding cancer care in Africa, and will endeavour to use this data to improve access to treatment, patient care, as well as patient outcome.

#### Addendum

The ACE-net aims to contribute to improving the knowledge about cancer cost of care and cost effectiveness in Africa. It aims to play a facilitating role for the advancement of cancer-related health economics and policy-making capabilities on the African continent. The ACE-net resorts under AORTIC and will promote and work on an interdisciplinary and interprofessional level. Through its programmes, the ACE-net will assist African countries in building capacity, providing education and training, and promoting national and international collaboration in the field of cancer health economics with learning and research institutions from other countries.

#### Vision

The vision of this network is to be an international health economics group focused on how to get better value cancer care in Africa, cost effectiveness, and cost of cancer care in Africa. It is in alignment with the AORTIC vision in creating awareness of the extent of cancer in Africa and to ensure that programmes related to cancer evaluation, cost, and research are firmly on the continent’s agenda.

#### Mission

The mission of this group is to evaluate the cost of cancer care and cost effectiveness on the African continent as well as to look for ways to get better value cancer care in Africa, to contribute to increase public and government awareness and support policy makers and civil society in our fight against the disease.

#### Core activities

The networking group strives to engage fully in the core activities of research and development, teaching and training, service rendering, and collaboration.

Its specific objectives are summarised as follows.

#### Research and development

The group will focus on health economics research on cancer (adult and paediatric) in all African regions.

#### Teaching and training

A spectrum of training modalities aimed to build up capacity will be included in the core activity of the ACE-net.

Training and mentoring researchers and research workers, nationally and internationally, through several means, i.e. conferences, workshops, short courses, webinar, and other distance programmes in cancer economics/policy-related subjects.

Provide and facilitate training in cancer health economics and public health through online and other comprehensive e-learning resources.

#### Service to community

Service to community will include: evaluation of cancer care costs and cost effectiveness of treatments and protocols, increasing awareness of public and government, and recognition of cancer and cancer-related pathology as a priority on the African continent.

#### Collaboration and other

The ACE-net will partner with other organisations and institutions African or international, which have overlapping or complementary interests and activities. These partnerships may entail:

Joint conduct of projects. Partner organisations may contribute to different projects and programmes of the ACE and vice versa.

Other objectives include project management (contracts, regulatory support, monitoring, coordination of projects, and programmes in African countries, which need support, training, guidance, etc).

#### Organisational structure

The ACE-net resorts under the AORTIC governance with meetings every two years together with AORTIC conferences.

#### Management Committee Composition:

The Management Committee will comprise the following members:

- the chair;
- a representative from each African region;
- consultant specialists in the field (health economics, policy makers, but not more than one for each);
- international consultants (not more than three).

### Breast cancer

In the Free Communication Session of Abstracts II, held on Friday, 22 November 2013, Dr B Yonas of Ethiopia made a presentation titled: ‘Breast cancer subtypes in Ethiopia: results of 46 breast cancer biopsies 2006–2010 from Addis Ababa University Hospital in Ethiopia’. The presenter remarked that breast cancer was an emerging burden of disease worldwide and that most scientific knowledge was derived from research done at academic institutions in Europe and North America, and that recent surveys showed fundamental differences concerning the presentation of the disease in African countries, e.g. more triple-negative cases. Of 1273 breast cancer cases diagnosed between 2006 and 2010 at the institution, 46 specimens were stained for oestrogen/progesterone receptor, HER2, and basal markers to determine their biologic subtypes. Clinical information was obtained from the patient files. Distant metastasis-free survival was available with a median follow-up time of 23 months. Immunohistochemistry (IHC) was done using an automatic staining machine. Median age was 40 (22–68 years), and mean number of children 3.8, median tumour size was 6.4 cm. Stages I and II, and III were seen in 20% and 41%, respectively. The majority of tumours was ductal and grade 2 (60%). Of 40 tumours with adequate IHC staining, 48% were hormone receptor-positive, and 29% were HER2-positive. Twenty-eight per cent (28%) had luminal a phenotype (HER2-negative), and 20% luminal B phenotype (HER2-/Ki67-positive). In hormone-receptor negative patients there were 22% HER2-positive and 30% triple-negative tumours. Median follow-up was 18.4 months. Kaplan–Meier estimates of distant metastasis-free survival after two years were 68%. The presenter admitted to the limitations of the study being the first of its type from Ethiopia, and given the small sample size as well as other technical limitations. He highlighted the fact that about 50% of patients could benefit from hormonal therapy and that almost 30% were of HER2-positive, features that were predictive for therapeutic options. Responding to questions about the relatively low frequency of triple-negative breast cancer (TNBC) in the sample, as compared with findings in West Africa, where up to 50% of breast cancer have been published as triple-negative, the presenter suggested that it might be a regional feature of the disease, perhaps, indicating regional variability of breast cancer subtypes in Africa.

In a poster presentation on 24 November 2013, Edmund Mounir Der reported on a study co-authored by Jehorant T Anim, Richard K Gyasi, Tettey Yao, Sophia Merajver, and Lisa Newman of Korle-Bu Teaching Hospital, Ghana, and University of Michigan, United States. The goal of the study was to evaluate the breast cancers diagnosed in one of the largest health care facilities in western Africa, and to compare the frequencies as well as risk factors for TNBC versus non-TNBC. They reviewed all breast cancer cases that had immunohistochemical assessment (Novolink detection system), in 2010. There were 223 breast cancer cases with the median age of 52.4 years. Most of the patients had palpable tumours (larger than 5 cm in diameter). More than half were TNBC (130; 58.3%). Similar frequencies of young age at diagnosis, stage at diagnosis, and tumour grade were observed among cases of TNBC compared with cases of non-TNBC.

In a Free Communication session on 22 November 2013, Dr Sarah Rayne presented a paper on ‘Young and Aggressive? A Comparative Study of Tumour Characteristics in Racial Groups of Breast Cancer Patients in Johannesburg’, based on a study from University of the Witwatersrand, South Africa, in which the global racial disparity in breast cancer survival and the belief that black women tend to have more advanced and aggressive disease was evaluated. The aim of the study was to determine whether tumour biology varied significantly with race. Over a period of one year, findings in consecutive patients from an uninsured population diagnosed with an invasive or *in situ* breast malignancy were reviewed and analysed. Data from radiological reports and histology were recorded in addition to demographics including age and race. Tumour characteristics between races were compared, particularly with reference to black patients. Of 334 patients with a new diagnosis of breast malignancy, 309 had adenocarcinoma, including 292 invasive ductal carcinomas, 12 lobular carcinomas and 13 patients had ductal carcinoma *in situ*. Other malignancies were five lymphoma and seven sarcoma patients. The median age at diagnosis was 55. 65.3% (218) of patients presenting with a breast malignancy were black. The remaining 116 patients were white (17.1%), Asian (6.9%), coloured (5.7%), and unknown (5.1%). In a comparison of invasive adenocarcinoma patients with known race only (*n* = 314), 86 patients with malignancy were below 45 years, including 32.8% of black patients and 18.7% of non-black patients (*p* = 0.0378); 84 of 218 black patients (38.9%) and 28 of 96 (29.2 %) of non-black patients had a grade 3 tumour (*p* = 0.1789). Overexpression of HER2 receptors was found in 63 (20.1%) of all invasive adenocarcinomas, including 42 (19.3%) of black patients and 21 (21.9%) of non-black patients (*p* = 0.7264); 52 (16.6%) patients were diagnosed with triple-negative malignancies, including 17.0% of black patients and 15.6% non-black (*p* = 1.000). Dr Rayne concluded that the experience in her institution suggested a relationship between race and a younger age at presentation, but did not support a link between race and biologically aggressive tumours, with none of the three surrogate markers for aggressiveness being significantly found to be more common in black patients seen at the institution.

In another Free Communication Abstracts II presentation, Dr Hannah Simonds of Groote Schuur, South Africa, made a presentation titled ‘Breast Cancer Tumour Subtypes in A Single Institution in South Africa’. The aim of the study was to explore the reported linkage between aggressive triple-negative disease and Afro-American heritage based on experience in a Western Cape breast carcinoma cohort. The study was a retrospective review of patients attending the oncology unit at Groote Schuur in 2012. Data collection included demographics, including age and race, as well as tumour characteristics, including ER, PR, and HER2 status, but excluding Ki67, which was not recorded at the institution. Of the 400 patients on whom records were available, luminal A comprised the majority of patients at 39.5%, luminal B 31.8%, TNBC 16.3%, and HER2-enriched 12.5%. The median age of the cohort was 56 years and the majority of patients, 277 (69.3%) were mixed race, and 83 (20.8%) were black. There was no significant association between race and TNBC molecular subtype; however, there was a trend to an increase of the subtype in the black race patients (18.1%). Dr Simonds concluded that the incidence of TNBC in this cohort was similar to those reported in international literature. Due to the small Caucasian population at the institution, it was not possible to draw definitive comparative conclusions regarding race and incidence of high-risk molecular subtypes.

In the Free Communication of Abstracts IV session, held on 23 November 2013, Dr Olufunmilayo Olopade presenting on behalf of Dr Dezheng Huo, both of the University of Chicago, on the topic titled: ‘Genome-Wide Association Studies of Breast Cancer in Women of African Ancestry Identifies Novel Susceptibility Variants’, stated that while in the past five years, a number of genome-wide association studies (GWAS) had identified more than 70 breast cancer susceptibility loci, most of the susceptibility single nucleotide polymorphisms (SNPs) were discovered and validated in Caucasian women. The aim of the study was to identify additional novel breast cancer susceptibility variants in women of African descent, including Nigerians, African Barbadians, and African Americans. A total of 1657 cases and 2029 controls were genotyped using the Illumina HumanOmni2.5 array. In total, 2,116,365 SNPs were genotyped and passed the extensive quality control. Of the 27 previous GWAS-identified loci in women of European or Asian ancestry, only four loci (5p15.33/TERT, rs10069690; 6q25.1/ESR1/C6orf97, rs9397435; 14q31.3/GALC, rs4322600; and 16q12/TOX3, rs3104793) were observed to be significantly associated with breast cancer risk in women of African descent (*p* < 0.05). In addition, several novel loci for breast cancer, including 5q12.3, 5q15, 8q24.3, 9p22.3, 12p12.1, 13q31.1, and 14q24.2 (*p* < 0.00001) were identified. Further studies in women of African ancestry were ongoing to validate these novel breast cancer susceptibility loci. In conclusion, Dr Olopade stated that the study highlighted the importance and necessity of conducting breast cancer genetic studies in diverse populations. To reliably apply findings of genotype–phenotype associations based on common low-penetrance alleles to breast cancer risk prediction in the clinic, further replication, and validation of GWAS findings using women of African ancestry are warranted.

### Breast cancer therapy

At the 23 November 2013 session on ‘Breast Cancer II: Focus on Oncology Therapy and Access to Care’, Dr Ahmed Elzawawy presented a paper on the changing trends in the management of breast cancer at the Suez Canal University, Alsoliman Center in Port Said, Egypt. A programme of free access to chemotherapy was commenced at the centre in 1984, while in 1994, radiotherapy became available at the centre through a charity facility, the Alsoliman Radiotherapy Centre. The later has progressively improved to reach its current status. The centre offers comprehensive management free of charge, for all citizens. Over the stated period of time, the centre has witnessed a decline in presentation with advanced disease. The mean time from onset of symptoms to presentation has declined from 18, 8, 3, 1, and 1 month, respectively, in 1987, 1989, 1999, 2007, and 2012. While in 1984, no patient was considered a candidate for breast conservation, 21.8% of 151 patients were found suitable for it in 2012. Five-year survival rates increased from 35% in during 1984–1988 to 86% during 2003–2007. In the period 2007–2009, local recurrence rate at three years was 0.7% among patients with mastectomy and 0.3% after breast conservation. These rates are among the lowest in the world. Dr Elzawawy described a ‘win-win scientific initiative’ (www.icedoc.org/winwin.htm) aimed at increasing affordability of ‘better value care’ tailored to different communities by exploring scientific approaches. He advised that early detection programmes would be frustrating for patients and health authorities in the absence of access to appropriate care.

At the Free Communication of Abstracts IV session, held on 23 November 2013, Dr Verna Vanderpuye of the National Radiotherapy Centre, Accra, Ghana, presented a paper titled: ‘A pilot survey of breast cancer management in Africa’. This was an anonymous online survey of breast cancer management among AORTIC members, using a 42-question structured questionnaire in both English and French. Twenty members from 19 facilities in 14 countries responded. Most of the responders were oncologists. Twelve belonged to a multidisciplinary breast cancer team. Radiotherapy equipment was available in seven facilities but four facilities had equipment down time at least once a week. Commonly available chemotherapy drugs included methotrexate, cyclophosphamide, fluorouracil, anthracyclines, vincristine, and taxanes, whereas trastuzumab, vinorelbine, and gemcitabine were only available in a few facilities. For diagnostic purposes, core needle biopsy was available in 16 facilities, mammogram in 17, computerised axial tomography in 15, magnetic resonance imaging in 11, and bone scan in nine. IHC was available locally in eight facilities, outside hospital but within the same country in seven, and outside of country in four. In 13 facilities, axillary node dissections were regularly performed. Neoadjuvant chemotherapy was the most common initial therapy for locally advanced breast cancer in 13 facilities. In three facilities, receptor status did not affect hormone treatment decision. Dr Vanderpuye concluded that the survey suggested that AORTIC members continued to make gains in breast cancer management by providing access to multidisciplinary breast cancer care but lacked important resources, such as immunohistochemical facilities and reliable radiotherapy services. Focus on in-country training and improvement of health care delivery in relation to cancer care services were urgently needed to provide quality breast cancer treatment in Africa. She explained that the oncologists, who replied to the pilot survey, were ‘clinical oncologists’, i.e. those trained to give general oncology service to patients, including radiation therapy, chemotherapy, and support care. When asked about effort to train physicians specialising in the management of systemic disease of cancer, such as medical oncologists, given the common late presentation in Africa, she replied that this was like ‘opening a can of worms’, apparently referring to ongoing controversy about the idea.

### Haematological malignancies

#### Myelodysplastic syndrome

In a presentation at the Haematology session on 23 November 2013, Dr Anthony Oyekunle presenting on behalf of his colleague, Dr MA Durosinmi of the Obafemi Awolowo University, Ile-Ife, Nigeria, sought to bring into focus the problems associated with managing myelodysplastic syndromes (MDS) in Africa in the face of inadequate diagnostic options and challenges of classification and provision of appropriate therapy. He observed that MDS are not uncommon in Africa, but that the clinical features are similar to published reports from other parts of the world. Diagnosis is limited to morphologic examination of peripheral blood and marrow cells, while facilities such as cytogenetics and immunophenotyping of tumour cells are very limited, especially in the majority of SSA countries. FAB classification is the norm in most of the centres. The more all-encompassing WHO classification technique was limited to a few centres in the North and South Africa, thus making stratification of patients into risk groups based on International Prognostic Scoring System impossible. Dr Durosinmi expressed the hope that efforts could be made to upgrade levels of haematology/pathology laboratories in SSA to high-tech standards with facilities for IHC, immunophenotyping, cytogenetics, and molecular pathology techniques, so as to enable better characterisations of haematological neoplasia, including MDS.

#### Chronic myeloid leukaemia

In his presentation at the Free Communication Of Abstracts II of 22 November 2013, titled ‘Survivorship in Nigeria Patients With Chronic Myeloid Leukemia: A study of 527 Patients Over 10 years’, Dr Anthony Oyekunle of the Obafemi University Teaching Hospital, Ile-Ife, Nigeria, observed that the advent of the tyrosine kinase inhibitor (TKI) had markedly changed the prognostic outlook for patients with Ph+ and/or BCR-ABL1+ chronic myeloid leukaemia (CML). The study was designed to assess the OS of Nigerian patients with CML on imatinib therapy. All CML patients treated in the institution on imatinib from July 2003 to June 2013 were reviewed. The median age of the patients was 37 (range: 10–87) years, and the gender distribution was male/female = 320/207; 472 were in chronic, 47 in accelerated, and seven in blast phase; 442 patients are alive by June 2013, with median survival of 105.7 (95%CI, 91.5–119.9) months; and OS at one, two, and five years were 95%, 90%, and 75%, respectively, with the survival in CP being significantly better (*p* < 0.0001) compared with those in AP or BP (107.3, 74.7, and 53.7 months, respectively).

After ten years of follow-up, imatinib monotherapy continues to give impressive survival outcomes among Nigerian CML patients. However, the patients have no access to second line TKIs, possibly accounting for the reduced survival when compared with outcomes in Western populations. In the question period, Dr Oyekunle described several complications of hyperleucocytosis that was common at presentation, frequently associated with organ impairment, including vision and hearing loss, sometimes reversible by lowering of the white blood count.

In a poster presentation on 21 November 2013 titled ‘Unusual Presentations of Chronic Myeloid Leukaemia’, Dr Amma Benneh-Akwasi Kuma described a number of patients presented with hearing loss and priapism as unusual presentation of CML. They constituted 8.3% of the patients seen at the centre. These manifestations of hyperleucocytosis associated organ failure constitute a source of compromise of quality of life that could be prevented by early diagnosis.

In a poster presentation titled ‘Laboratory Diagnostic Review of Chronic Myelo-Proliferative Neoplasms at a Pathology Practice in Kenya’, made on 23 November 2013, Dr Ahmed Kalebi and Dr Ruchika Kohli outlined their experience in the investigation and diagnosis of myeloproliferative neoplasms (MPN). Including CML, essential thrombocythemia, primary myelofibrosis (PMF), and polycythemia vera (PV); 25% of the MPN cases were diagnosed on bone marrow trephine biopsy with 13% diagnosed with a bone marrow aspirate. BCR-ABL was frequently done in patients with suggestive CML on morphology to determine whether targeted therapy was indicated. Most of the patients with CML do have the BCR-ABL gene mutation—out of the 520 cases seen over the last three years, 74% (383) were positive for the mutation. The JAK2 mutation is less frequently requested—out of 41 cases, 12 were positive. They concluded that PCR for BCR/ABL translocation, and JAK2 mutation analysis have greatly improved the accuracy of evaluation of chronic myeloproliferative neoplasms (CMNs), while availability of bone marrow trephines have also contributed to better diagnosis of PMF.

### Cancer of the cervix

Dr Nathalie Broutet of the WHO, Geneva, Switzerland, who co-chaired the Cervical Cancer Prevention I session on 21 November 2013 informed the meeting that the WHO recently issued recommendations on the use of a ‘screen and treat’ approach using visual inspection with acetic acid (VIA) for screening and treatment with cryotherapy. These recommendations are published in the new WHO guidelines for screening and treatment of precancerous lesions for cervical cancer prevention. It is expected that this new approach would bring about a reduction in the incidence of cervical cancer, where it is implemented. Since WHO works only on voluntary basis with member countries, the new guidelines will be provided through the regional office, e.g. AFRO Headquarters for African countries. The new guidelines would be a simplification of the algorithm of care in resource poor settings. It is expected to avoid loss to follow-up among women with significant cervical findings. The impact of the new guidelines would need to be monitored. Other guidelines can be expected to follow, especially, because newer methods for disease detection are being developed, which may impact on future guidelines.

In the Cervical Cancer Prevention Session II, Dr Lynette Denny of the University of Cape Town, Cape Town, South Africa, in a presentation titled: ‘Training Human Resources in the Context of National Roll-Out of Cervical Screening’ explained that establishing a system for cervical cancer screening was complex and required resources at multiple levels to be effective and that the standard routine screening methods, which had been cytology based, required a mechanism for taking Pap smears, having them delivered to a laboratory, interpreted, the result sent back to the primary clinic or patient and women with abnormal smears then recalled for colposcopy, followed by histological assessment, treatment, and follow-up. She pointed out that where applied correctly, this approach, which was standard in resource rich parts of the world, reduced the incidence of cervical cancer significantly, but that developing countries had no resources to establish sustainable screening programmes of this nature, due to the lack of robust health care infrastructure and competing health care needs. With modern approaches using different screening tests, such as HPV DNA testing or VIA, along with ‘the screen and treat’ approach, it was envisioned that a less complex infrastructure would be required. Women would still need to be educated and encouraged to go for screening. Facilities for screening and training of personnel would still be required, and that for subjective tests like VIA, training would need to be ongoing with some form of reliable quality control in place. In addition, treatment needed to be carefully monitored for effectiveness and women would need to be followed up to ensure eradication of disease. Even ‘screen and treat’ would require training at multiple levels and should not be seen as a ‘soft option’ compared with the complexity of cytology-based screening programmes.

Similar views were expressed by Dr Z Mike Chirenje of University of Zimbabwe, College of Health Science, Zimbabwe, who in his abstract indicated that VIA allowed detection of pre-cancer lesions with sensitivity of about 75%, and that cryotherapy treatment could be offered immediately if a lesion was well demarcated. Nurse practioners could be trained to offer VIA and treatment with cryotherapy and that many countries in SSA had embarked on demonstration projects that would allow future planning for scaling up programmes. He suggested that each country must have a dedicated budget to support cervical cancer screening with adequate funds to train manpower that would sustain screening and treatment of CIN.

In the Cervical Cancer Prevention Session II, Groesbeck Parham of the Centre for Infectious Disease Research in Zambia made a presentation titled:‘100,000 Women Screened Through the Cervical Cancer Prevention Programme in Zambia’ in which he described Zambia’s response to the heavy national cervical cancer burden, whereby the Ministry of Health, University Teaching Hospital and Centre for Infectious Disease Research in Zambia, established a Cervical Cancer Prevention Service Platform using digital cervicography in cervical cancer screening services. Between January 2006 and June 2013, 101,106 women were screened for cervical cancer through the programme. The median age of women screened was 32 years (interquartile range: 26–39 years). 26,568 (26.3%) women were HIV-infected; 29,616 (29.3%) did not know their HIV sero-status and were offered HIV testing at the time of cervical screening. Of the 101,106 women screened, 19,093 (20.2%) were VIA screen positive. Of those that screened VIA positive 11,472 (60.1%) underwent cryotherapy and 3355 (17.6%) underwent either electrosurgical excision (‘see and LEEP’) or punch biopsy. Among 3355 women with a histologically confirmed diagnosis, 1688 (50.3%) had benign or low-grade cervical lesions, 905 (27.0%) had high-grade cervical lesions, and 762 (22.7%) were diagnosed with invasive cervical cancer. He concluded that the digital cervicography-based cervical cancer screening and treatment programmes were effective and scalable in resource-constrained settings like Zambia.

### Genetics of oesophageal cancer

In a poster presentation titled ‘Nat1 and Nat2 Genetic Polymorphisms and Interaction With Environmental Risk Factors on Susceptibility to Oesophageal Squamous Cell Carcinoma in South Africa’, Dr Marco Matejcic of Cape Town, South Africa, on 23 November 2013, reviewed the possible role of polymorphisms in the NAT1 and NAT2 loci and their interaction with environmental risk factors on susceptibility to oesophageal cancer in black and mixed ancestry South Africans; 732 oesophageal cancer patients and 768 healthy controls were genotyped for the NAT2 slow acetylator alleles (G191A, T341C, G590A, G857A) and the NAT1*10 allele (T1088A, C1095A), and the acetylation phenotype was inferred by the genotyping data. Significant differences in the distribution of NAT genotypes and acetylator phenotypes between cases and controls were tested for using the Pearson’s chi-square test. Logistic regression analysis was used to test for gene–environment interactions with regard to oesophageal cancer risk. The G191A variant (NAT2*5 allele) was associated with reduced risk of oesophageal cancer among mixed ancestry individuals (OR = 0.68; 95%CI = 0.52–0.88; *p* = 0.004). NAT1 and NAT2 acetylation phenotypes were not independently associated with oesophageal cancer risk in both population groups. However, exposure to tobacco smoke increased the risk only among NAT2 slow and intermediate acetylators in both black (OR = 2.76; 95%CI = 1.69–4.52; *p* < 0.0001) and mixed ancestry population (OR = 10.1; 95%CI = 3.54–29.11; *p* < 0.0001). The alcohol-related risk was present only among mixed ancestry individuals carrying NAT2 slow and intermediate genotypes (OR = 2.77; 95%CI = 1.38–5.58; *p* = 0.004). NAT1*10/*10 genotype was associated with a protective effect from tobacco smoke exposure in the black population (OR = 3.41; 95%CI = 1.95–5.96; *p* < 0.0001) and from alcohol consumption in the mixed ancestry population (OR = 3.41; 95%CI = 1.70–6.81; *p* = 0.001). Dr Matejcic concluded that NAT1 and NAT2 acetylation polymorphisms might have an important role in modifying the interaction between environmental risk factors and oesophageal cancer risk in black and mixed ancestry South Africans.

### Viruses and cancer

Making a presentation at the Viruses and Cancer session on 24 November 2013, Dr R Newton of the United Kingdom sought to explain the high incidence of Kaposi’s sarcoma in parts of SSA. He presented data showing that KSHV seroprevalence was associated with malaria and hookworm infection, and that KSHV is shed in saliva, whereby males are more likely to shed the virus in saliva than females. The relevance of this to the known gender related differential frequency of KS was not stated.

### Pathology

At the Pathology Plenary session, held on 22 November 2013, Dr Shahla Masood of the University of Florida, College of Medicine, Jacksonville, Florida, speaking by video link on the topic of ‘Pathology as the Core Foundation for Breast Care’, spoke about the role of the pathology in disease oriented teams, such as breast cancer care team. With the recent worldwide interest in establishment of breast centres offering integrated services via a multidisciplinary approach, the role of pathologists has become more conspicuous. As members of the breast care teams, pathologists are now actively participating in breast tumour conferences and in treatment planning of breast cancer patients. Recognised as the foundation of high quality breast health care, many societies have established guidelines for breast pathology reporting and have endorsed the role of pathologist as partners in breast care. She described pathology as the study of human illness, involving the morphologic and biologic recognition of abnormalities that are associated with a disease. Breast pathology represents an excellent example of this concept. By providing diagnostic information and by characterising the biologic behaviour of a breast lesion, a pathologist plays a critical role in a patient’s life. Any mistake in this exercise is associated with serious consequences. In addition, there are many unresolved issues in breast pathology, which contribute to our limited understanding of the biology of breast cancer, variability in diagnostic criteria, and significant diversity in breast cancer management and therapy. Furthermore, breast pathology has remained an under-recognised discipline among the public and some health care providers, and its importance in diagnosis and disease management is not fully realised. To better serve patients, particularly medically underserved women and those living in countries with limited resources, emphasis needs to be placed on effectively using the talent and expertise of pathologists around the globe.

Speaking at the Pathology Plenary session on 22 November 2013, on the presentation titled ‘Pathology Diagnostics in Sub-Saharan Africa: The Glorious Past, Current Status and Recommendations for Salvaging the Future’ Dr T Abisogun Junaid of the College of Medicine, Kuwait University, Kuwait City, Arabian Gulf, described the advent of health care services to SSA with European colonisation of SSA. With this came missionary doctors and physician pathologists, who set up health centres that grew into general, regional, and teaching hospitals of newly established medical colleges such as those of University of Makerere in Kampala, Uganda, the University of Ibadan, Ibadan, Nigeria, and the University of Khartoum, Khartoum, Sudan. Morbid Anatomical, light microscopic, and other basic laboratory techniques were usefully employed to map out disease patterns, establish research units, and correct misconceptions about disease occurrence and causation. Additionally, a handful of young African physicians and technicians were recruited into pathology. Scholarly publications from these centres established that cancer was as common in SSA as in parts of Europe but were of different patterns and histopathological subtypes. Entities such as Burkitt’s lymphoma and endemic Kaposi’s sarcoma were highlighted and their possible environmental causes discussed. Technological advances that have revolutionised pathologic diagnostics in the last four decades, and which have offered Pathology a historical opportunity, have coincided with the post-independence period of chaos, military dictatorships, conflicts, unplanned expansions, and ‘brain drain’ in SSA. Consequently, only a few laboratory services in SSA today have expertise in, or facilities for IHC, FISH/CISH, PCR, or DNA microarrays. Adequate staffing and upgrading of existing laboratory facilities would require political commitment, rational planning, judicious use of limited resources, and a re-evaluation at the specialty itself.

Dr Mary Gospodarowicz of the Princess Margaret Hospital, University of Toronto, Toronto, Canada, and President of UICC, speaking at the Pathology Plenary session on the topic of ‘Cancer Staging: A Fundamental Element of Cancer Control’, stated that the purpose of staging is to aid the clinician in the planning of treatment, to give some indication of prognosis, to assist in evaluation of the results of treatment, to facilitate the exchange of information between clinicians and treatment centres, to contribute to the continuing investigation in cancer, and to support cancer control activities. The TNM classification, on which she has collaborated with others at UICC, is a gold standard for prognostic judgment because it has a powerful correlation with outcomes and is required for treatment decisions. All clinical practice guidelines refer to stage, and the stage is a frequent entry criterion as well as stratification variable in clinical trials. Stage is also used in broader cancer control environment in planning for services and stage shift is an early indicator of the efficacy of screening programmes.

However, since the TNM classification is a descriptor of the anatomic disease extent it does not describe the tumour biology domain nor does it fully predict treatment response and needs to be supplemented by tumour profile characteristics that indicate qualitative features of the disease in addition to its extent. Clinical stage describes pre-treatment extent of disease based on assessment with imaging, biopsy information, and so on, while pathologic stage is determined at surgery and involves histopathologic examination of the entire tumour sufficient to determine the highest possible T and N categories. While initial treatment recommendations are based on the clinical stage, the recommendations for adjuvant therapy are mostly determined by pathologic stage. Synoptic reporting of tumour pathology includes the designation of the pathologic stage. Recording of cancer stage in hospital or population-based cancer registries greatly enhances their value, in assessing the value of screening programmes, informs resource allocation, evaluates compliance with treatment guidelines, compares survival trends, enhances cancer control, and should be standard. Dr Gospodarowicz indicated that another use of staging in cancer population study is to demonstrate the effect of cancer control in form of reversing the frequency of late stage to earlier stages, as a sign of more effective community cancer control, which may precede the observation of survival improvement. Thus, work in cancer registration should go hand-in-hand with cancer staging.

In his presentation on the topic ‘The WHO classification of lymphomas: can Africa really cope?’ Dr Ahmed of the Pathologists Lancet Kenya Limited, Nairobi, Kenya, highlighted the deficiencies in the practice of pathology in Africa that limit the ability to affect the guidelines of the WHO classification. He, however, cautioned that this should not limit a meaningful diagnostic service in lymphoma management on the continent. In subsequent discussion of his presentation, the point was made that African institutions should learn to make do with what they can afford (‘Cut your coat according to your size’), rather than waiting until they are able to ‘catch-up’ with the ‘gold standards’ of the developed world.

### Radiotherapy

In her presentation titled ‘Quality Assurance in Radiation Therapy’ on the importance of radiotherapy in cancer management, Dr Mary Gospodarowicz, President of UICC, and Professor of Medicine at Princess Margaret Cancer Centre, University of Toronto, Canada, described radiotherapy a critical ingredient of comprehensive cancer treatment and that anywhere from 40% to 50% of all cancer patients would benefit from receiving radiotherapy in the course of their illness. Radiotherapy can be used as a sole curative therapy, in combination with surgery and/ or chemotherapy as part of the initial curative treatment approach, in the management of recurrent disease, and as a powerful tool for palliation. Currently, the supply of radiotherapy services falls short of demand in many parts of the world. About two-thirds of the world lacks radiotherapy capability, including many high-income countries. However, the shortage is so pronounced in low- and middle-income countries, especially in Africa, that it precludes radiotherapy from being considered as a part of cancer management. Fiscal constraints and the pressure to treat as many patients as possible may lead to cost cutting initiatives, which in turn may result in compromised staffing levels, limited facilities, and less attention to quality. The framework for quality of radiation therapy must include consideration of appropriate infrastructure including facilities, equipment (hardware and software), people with skills, and credentials. Once in place, the framework should include regular quality assurance processes for equipment. Monitoring of medical decisions in patient assessment and planning, physics and therapy quality assurance of treatment plans, and peer review of final plans, are further steps in securing safe planning and delivery of RT. Modern techniques (IMRT and IGRT) require additional sophistication of radiotherapy quality assurance. Dr Gospodarowicz stated further that UICC had approved effort to determine the global gap in radiotherapy and the organisation is now better positioned to help in the process of provision of radiotherapy to needy parts of the world. A global task force has now been established to help in determining the global need for radiotherapy, and that there is an open invitation for participation in the activities of the task force.

### Psycho-oncology and palliative care

Dr Jimmie C Holland of the Memorial Sloan-Kettering Cancer Center, New York City, United States, making a presentation on ‘What Makes People With Terminal Cancer Suffer?’ stated that over the years since she has been associated with AORTIC, she has observed changes in the Africans’ attitude towards cancer, and believes that the organisation has transformed the awareness to NCDs, and cancer in particular. She described her discipline as being concerned about bringing focus on the psychological, social, and cultural aspects of cancer. Late disease presentation, which is prevalent in Africa, is due to several factors, including cultural, lack of knowledge, spiritual, etc. It often leads to reactions from others that make the problems worse for the patients, e.g. abandonment of the patient by relatives, who are confused about the nature of the patient’s illness. Given the fact that the easiest access to care for most of the Africans is to the traditional healers, this frequently contributes to delays in presentation for orthodox care. Thus, a way has to be found to integrate the traditional services with those of orthodox care.

Cancer care has long focused exclusively on management of the cancer itself, rather than the psychological impact of the disease on the patient.

The word ‘distress’ has begun to be used to cover the several psychosocial issues that a cancer diagnosis raises, which are universal and cross cultural, including:

- dealing with the PHYSICAL symptoms of pain, fatigue, and treatment side effects;
- the PSYCHOLOGICAL effects of sadness and fears;
- the SOCIAL meaning for the family, for finances and the future;
- the SPIRITUAL, which involves the beliefs that bring comfort to the person;
- the EXISTENTIAL, which involves seeking the meaning of life in the face of death.

Each particular culture adds its own problems to distress. For example, the STIGMA attached to cancer, particularly in Africa, adds to the distress by making the person feel unacceptable, sometimes to family and others. It is these issues that psycho-oncology seeks to address as it brings attention to the care of the whole person.

Palliative care has as its goal to relieve ‘pain and suffering’. The management of ‘suffering’ requires effective communication, not only in terms of what is said or spoken, but how it is said or verbalised, including unspoken body language, culturally appropriate touch or look (eye contact during communication, for instance). Discussion of impeding death can be carried out in such a way that the focus is on how to make the best use of the time left rather the topic of death itself.

Faith is an important issue, especially in the African society with a background in spiritualism. These issues are increasingly recognised as being extremely important in the palliative care of patients. They must be included in developing an optimal quality palliative care programme.

### Dr Fiona Rawlinson, consultant in palliative medicine, Wales, UK, provides the following report of palliative care as presented at the Ninth AORTIC Conference

Palliative care was a significant part at AORTIC, delivered through plenary sessions, workshops and poster presentations. Developing practice and initiatives from many African countries were highlighted and the pivotal role of the African Palliative Care Association (APCA) clearly visible.

#### Palliative Care 1

Workshop palliative care 1 was chaired by Ann Merriman from Hospice Africa Uganda and Fatia Kiyange from APCA.

The workshop began with ‘What do we mean by palliative care?’ delivered by Fatia Kiyange (APCA/Uganda) on behalf of Dr Faith Mwangi-Powell. The central role of palliative care in delivering holistic care to the patient and their family was described with the key elements of physical, psychological, spiritual, and social domains, recognising death as a natural event and focusing of optimising quality of life. The need to ensure that decisions about care have patient involvement was highlighted.

Prof Yennu (USA) then followed and shared the findings of a number of studies conducted in USA demonstrating improved patient outcomes and quality of care with timely referral to palliative care services. Some of the discussion points centred round the fact that in some areas of the world there is debate over the most appropriate time of referral in a patient’s journey: should it be at the time of diagnosis, or when the last few weeks of life are apparent? A further area for debate is the name ‘palliative care’: does it reflect fully what care is provided, and is it a worrying term for some people, is ‘supportive care’ a more appropriate, and perhaps less daunting term for patients? However, it was also acknowledged that these are observations from a different health system where earlier diagnosis is more often achieved and within Africa, the need to improve on early diagnosis remains a priority for cancer care. In Africa, by the time the diagnosis is made, many patients have significant palliative care needs.

Dr Vanderpuye (Ghana) described the challenge in providing effective palliative care for patients with colorectal cancer in Africa. The newer agents, which have contributed to improved symptom and quality of life outcomes in some parts of the world, are not affordable or not available in many parts of Africa, which leaves patients with unmet needs that desperately need to be addressed.

Mark Lazenby (USA) described some collaborative work between Yale, USA, and Botswana depicting possible changes in place of death and the increasing numbers of deaths in hospital in an area in that country.

Tracy Brand (South Africa) completed the session with an engaging and informative description of the challenges of communication with children and their parents about cancer. This was an energising and practical approach for workshop attendees to follow with many hints and tips given. The need for aligning professionals’ communication with that of the child’s needs, at the appropriate development level and the need for respect for the child’s space was highlighted. ‘I don’t care what you think unless I think you care about me’.

#### Palliative Care 2

The afternoon workshop covered the palliative care issues of cervical, prostate, and breast cancer with contributions from Dr Ramondetta (USA), Dr Ali (Kenya), and Dr Mwebesa (Uganda): all illustrating the need for continued and developing palliative care skills for health care professionals looking after patients with these conditions. The burden of symptoms in all domains (physical, psychological, spiritual, and social) remains high, despite the advances in trying to diagnose the diseases earlier.

Dr Holland (USA) reminded us of the need to continue to establish levels and reasons for distress including the psychological, emotional, and spiritual burden of disease and Dr Ngoma (Tanzania) outlined the need for paediatric palliative care skills and how these can support the ongoing care of children with cancer.

#### Keynote sessions

Dr Ramondetta delivered the first keynote session ‘Placing patients humanity at the centre of our care’. This was a very powerful presentation asking all present to examine their own reaction to illness and existence in order to be able to deliver compassionate human-centred care. The challenge of managing technology during a patient consultation was also described; many senior professional were taught communication skills before the advent of computers, which now risk being a barrier and almost a ‘third person’ in the consulting room. A memorably and seeringly vivid video from USA was shown to a hushed conference hall depicting simply in film, music, and simple written text the possible thoughts and feelings of patients, relatives, and health care professionals as they moved around a hospital in the United States. This urged us all to remember the person behind condition or the professional and would make a powerful teaching aid for people training health care professionals across the world. (***Empathy: the human connection to patient care***. http://www.youtube.com/watch?v=cDDWvj_q-o8)

#### African palliative care and its contribution to the world

Dr Mwangi Powell then gave the second lecture outlining the powerful impact of the African Palliative Care Association in helping to develop and encourage sustainability of palliative care services throughout the continent. The approach has been strongly based on the WHO’s four-pillar public health model of palliative care provision: appropriate government policies (e.g. a national health, essential medicines, and education), effective drug availability, education of health professionals, and implementation of palliative care at all levels of health care provision. Significant developments have occurred since APCA’s establishment in all four areas including a key note meeting and subsequent consensus statement at the recent APCA/HPCA conference in Johannesburg in September 2013 where commitment was given from a number of countries towards establishing availability of pain relief and integration of palliative care into health systems.

#### Pain management workshop

A workshop on pain management then followed, supported by UICC and GAPRI. This opened with an overview on the advances in access and ability to strong opioids across Africa. Dr Ali (Kenya) and Dr Vanderpuye (Ghana) reported on projects that monitor and evaluate accessibility of strong opioids and highlighted that, although there have been some significant developments, the need to continue the work is still an imperative. Mention was made of the meeting in Johannesburg in September at the APCA/HPCA conference and the continued need for collaboration and development of strategies to ensure that pain control, a basic human right, is improved upon.

The second session described practical issues for meticulous quality in implementing the manufacture of oral morphine solution. In Uganda, this is a centrally organised system with stringent quality checks. In Nigeria, this has been implemented regionally and with similar quality control. These were interesting, thought provoking presentations inspiring hope for countries looking to develop their own sustainable morphine production for patients who need it for their pain control and breathlessness control. Helpful and practical issues were considered, such as cost-effective ways to source the components and resources needed. Throughout the session, there was ongoing discussion about integrating legislation about safe painkiller availability and administration into government policies.

#### Posters

Palliative care issues featured in the posters presented during each of the days. Symptom control and psychosocial, communication/spiritual issues were reflected in a number of posters from different African countries with complementing topics, such as traditional healers, symptom burden, and service delivery. Raising awareness of the need to support development palliative care skills through educational initiatives using newer technology, such as online programmes to deliver education to improve patient care also featured.

#### Summary

Palliative care had a strong presence at the AORTIC conference in Durban, complementing presentations and sessions covering cancer pathology, diagnosis, management, and care. The need to incorporate palliative care skills into oncology services was evident and the pivotal role of the APCA in helping to achieve this was evident.

### AORTIC’s continental cancer plan

In the closing session of the conference, titled ‘AORTIC’s Continental Cancer Control Plan’, Dr Issac F Adewole of Nigeria, Vice Chancellor of the University of Ibadan, Ibadan, Nigeria, and AORTIC President presented AORTIC plan to control cancer in the continent. He expressed the view that a major problem of cancer in Africa appears to be the widespread lack of knowledge about the disease, even among African physicians! He described a trip that he and Dr Lynette Denny, the AORTIC Secretary/Treasurer undertook to the AU Headquarters in Addis Ababa, Ethiopia, where most of the African diplomats were astonished to hear that cancer was a problem in Africa. The AORTIC plan for cancer control in the continent will focus heavily on advocacy and education. A number of cancers have been identified, including cervical, breast, prostate, liver, lung, etc. Countries and regions would identify which of these are more important for them. The emphasis would be on prevention and early detection as well as treatment.

## Conclusion

The main take-home messages of the Ninth AORTIC International Conference on Cancer in Africa are as follows:

- Africa is finally awakening to the reality of cancer.
- Cancer control is possible in the resource-poor settings of the low- and middle-income countries at reasonable cost. This applies to 70%–80% of the population of the world.
- Modest approaches in cancer control have the potential to have a major impact on cancer morbidity, mortality and suffering, not only in countries with limited resources, but also in countries with high resources where millions have limited access to care.

Table 1 shows AORTIC conference statistics, 1983–2013.

## Figures and Tables

**Table 1.** International meetings of AORTIC on cancer in Africa

Date	Host site	No. of participants	No. of nations participating	No. of posters presented	No. of abstracts submitted	No. of oral presentations
22–23 July 1983	Lome, Togo	24	14^a^	N/A^*^	N/A^*^	N/A^*^
11 November 1985	Brazzaville, Congo^b^	61	?	0	0	?
30 October–3 November 1989	Kinshasa, Zaire	≈100	28	0	80	70
1990–2000	Years of inactivity/dormancy					
6–10 October 2003	Accra, Ghana	?	?	?	?	?
14–16 November 2005	Dakar, Senegal	200	32	?	165	4^**^
25–28 October 2007	Cape Town, South Africa	400	46	116	141	18
11–14 November 2009	Dar Es Saalam	600	50	118	272	22
30 November–3 December 2011	Cairo, Egypt	300	51	242	433	53
21–24 November 2013	Durban, South Africa	900	70	200	450	72^***^

* N/A—Business meeting.

** Four keynote presentations.

*** Including six plenary presentations.

a Including Benin, Cameroun, Republic of the Congo, Ivory Coast, Kenya, Liberia, Malawi, Mali, Nigeria, Senegal, Sweden, United State of America, Uganda, and Upper Volta (now renamed Burkina Faso).

b World Health Organisation Regional Headquarters for Africa.
